# Effects of Various Penetration Enhancers on Penetration of Aminophylline Through Shed Snake Skin

**DOI:** 10.17795/jjnpp-12904

**Published:** 2014-02-20

**Authors:** Maryam Kouchak, Somayeh Handali

**Affiliations:** 1 Department of Pharmaceutics, Faculty of Pharmacy, Ahvaz Jundishapur University of Medical Sciences, Ahvaz, IR Iran; 2 Nanotechnology Research Center, Ahvaz Jundishapur University of Medical Sciences, Ahvaz, IR Iran

**Keywords:** Aminophylline, Tauroglycocholic Acid, Lauric Acid, Ethanol

## Abstract

**Background:**

Cellulite is the accumulation of subcutaneous fat and connective tissue in tights and buttocks. Xanthines, such as aminophylline, are used as phosphodiesterase inhibitors, and are also adenosine receptor antagonists.

**Objectives:**

The aim of the present study was to characterize *in vitro* aminophylline transdermal absorption through shed snake skin, and to investigate the absorption enhancing effect of various enhancers.

**Materials and Methods:**

Aminophylline gels were prepared using theophylline and ethylenediamine as raw materials of aminophylline, hydroxypropyl methyl cellulose (HPMC) F4M as gelling agent, and propylene glycol as a co-solvent. Sodium tauroglycocholate (STGC) (100, 200, and 500 μg/mL), lauric acid (1.7 and 15%), and ethanol (60%) were added as enhancers. In vitro percutaneous absorption experiments were performed on snake skin using Franz diffusion cells. Flux (J), permeability coefficient (P), and enhancement factor (EF) for each formulation were calculated.

**Results:**

The results indicated that all of enhancers significantly enhanced drug permeability. This effect was decreased by increasing the concentration of STGC; in contrast, by increasing the concentration of lauric acid from 1.7 to 15%, EF was enhanced Although ethanol (60%) and STGC (100 μg/mL) showed the highest EFs, the effect of ethanol on drug permeability appeared with a lag time.

**Conclusions:**

According to the findings, type and concentration of penetration enhancers can effect on transdermal permeation of drug.

## 1. Background

Cellulite is defined as topical changes in the skin of thighs and buttocks and described as orange peel or cottage cheese. Cellulite may also occur in breasts, upper arms, and lower abdomen. These changes are most commonly observed in women over 35 years, and 85% to 98% of post-pubertal females display some degrees of cellulite. It is prevalent in women of all races and may occurr due to hormonally mediated fat deposition, reduced venous return, reposition of capillary vasculature by fat lobules, and deposition of proteins around clumped fat lobules (1-3). Different methods have been used for treatment of cellulite, including physical and mechanical treatments (liposuction, phosphatidylcholine injections, ultrasound, laser-assisted liposuction, and infrared light), pharmacological agents (such as xanthines, retinoids, and lactic acid) and herbal products (1, 3-8). Recently, aminophylline and retinoids have been critically evaluated (1). Aminophylline is a xanthine derivative. When theophylline is combined with ethylenediamine in an anhydrous alcohol, it forms the compound aminophylline employing as a phosphodiesterase inhibitor that stimulates beta-2 agonist receptor activity increasing the levels of cyclic AMP (cAMP). It has been suggested that aminophylline inhabits the activity of certain hormones that cause fat storage followed by release of intercellular triglycerides. It seems that aminophylline blocks the receptors for these hormones located on fat cells (1, 2, 9). The transdermal method is used as one of systemic drug delivery routes with several advantages such as avoidance of first-pass effect, ease of use, ability to control drug delivery for a longer time compared to usual gastrointestinal transit of oral form, and is associated with patient compliance. The major limitation of this route, however, is difficulty of drug permeation through skin. The major barrier for drug transportation through the skin is stratum corneum. Stratum corneum is the outer layer of skin, which is the actual physical barrier to most substances contacting to skin. The permeation of drug through skin can be accelerated by both chemical and physical methods (10-12). There are several physical enhancements such as iontophoresis, electroporation, ultrasound, microporation, and micro-needles (13). Chemical penetration enhancers act through different routes including disruption of stratum corneum lipid, interaction with intercellular protein, and improved partitioning of the drug, co-solvent, or co-enhancer into the stratum corneum (14). Various chemical enhancers have been evaluated for transdermal absorption such as fatty acid (e.g. oleic acid, lauric acid, alkanoic acids, and capric acid), alcohols (e.g. ethanol, 1-octanol, 1-hexanol, 1-decanol, and lauryl alcohol), sulfoxides and similar compounds (e.g. dimethyl sulfoxide (DMSO), dimethylacetamide, and dimethylformamide), azones, cyclodextrins (e.g. 2-hydroxypropyl-ß-cyclodextrin), and surfactants (15, 16). Surfactants are used in pharmaceutical, cosmetics, and food products as wetting agents, adhesives, solubilizers, detergents, emulsifiers, and suspending agents. They can be classified into four main categories according to the presence of formally charged groups in the head; anionic (e.g. sodium tauroglycocholate), cationic (e.g. cetyltrimethyl ammonium bromide), nonionic (e.g. polyoxyethylene sorbitan monopalmitate), and amphoteric (e.g. *N*-dodecyl-*N*, *N*-dimethylbetaine) (15). Chemical penetration enhancers should be non-toxic, non-irritating, non-allergenic, have no pharmacological activity within the body, ideally work rapidly, and when removed from the skin, barrier properties return to normal status both rapidly and completely (12).

## 2. Objectives

The aim of the present study was to modify aminophylline transdermal absorption, using various skin permeation enhancers, including sodium tauroglycocholate (STGC), lauric acid, and ethanol.

## 3. Materials and Methods

Hydroxypropyl methyl cellulose (HPMC) F4M was bought from Colorcon Company, USA. Sodium tauroglycocholate (STGC) and lauric acid were obtained from Camlab Chemicals, England and Applichem GmbH, Germany, respectively. Propylene glycol, sodium sulfite, theophylline, ethylenediamine, monobasic potassium phosphate, and ethanol were bought from Merck, Germany. The snake skin was kindly donated by Razi Institute, Karaj, Iran.

### 3.1. Preparation of Aminophylline Gel

Aminophylline were prepared using theophylline and ethylenediamine as raw materials (Remington) (17). For preparation of aminophylline gel, HPMC F4M was used as gelling agent. Briefly, propylene glycol (as co-solvent), theophylline, and ethylenediamine were dispersed in distilled water under constant stirring by a paddle stirrer and heated to 800°C. Then, HPMC was added slowly. Compositions of base formulation are shown in Table 1. Sodium tauroglycocholate at concentrations of 100, 200, and 500 μg/mL was added directly to gel formulation. Lauric acid (1.7 and 15%) was dissolved in adequate amount of hot water and added to the formulation. Gel contenting ethanol as penetration enhancer was prepared according to Table 1 by using ethanol (60%) instead of water. 

**Table 1.** Composition of Base Formulation for Aminophylline Gel

Compound	Amount, %
**HPMC F4M**	3
**Theophylline**	1.7
**Ethylenediamine**	0.3
**Propylene glycol**	15
**Distilled water**	to 100

### 3.2. In Vitro Skin Permeation

Shed snake skin of *Vipera labatina* was used as model membrane. The skin was hydrated by immersion in water at 40˚C for 30 minutes before the experiments, and mounted in a Franz-type diffusion cell. One gram of each sample was applied on the shed snake skin. An aliquot of 3 mL of sample was withdrawn from the receptor compartment at 0.5, 1, 2, 3, 4, 5, 6, and 24 hours, and replaced by the same volume of distilled water at 37˚C to maintain the volume constant. The concentration of released drug was assayed at 272 nm using spectrophotometry apparatus.

### 3.3. Data Treatment and Statistical Analysis

The rate of drug permeation via skin can be determined using Fick’s first law (Equation 1-3). According to this law, the amount of drug (M) flowing through a unit cross-section area (S) of the skin and appearing in the receptor solution in time t is known as the steady-state flux, J:

Equation 1. dM/Sdt = J = DC_0_ K/h

Equation 2. P = KD/h

Equation 3. J = PC_0_

Where D stands for diffusion coefficient of the drug in the stratum corneum, h stands for diffusional path length or membrane thickness, K stands for partition coefficient of drug between stratum corneum and the vehicle, C_0_ stands for applied drug concentration assumed to be constant during the experiment, and P stands for permeability coefficient of drug in stratum corneum. The ﬂux, J, was determined using slope of steady-state portion of drug amount permeated divided by S versus time. The lag time values were determined by x-intercept of linear region at steady-state. The enhancement factor (EF) for each enhancer is calculated using equation 4.

Equation 4. EF= J_1_/J_0_

Where J_0_ and J_1_ stand for permeation rates in absence and presence of enhancer, respectively (14, 18-20). All experiments were carried out in triplicate and expressed as Mean ± SD. Statistical data analysis was performed using one-way ANOVA.

## 4. Results

The value of flux (J), and enhancement factor (EF) are shown in Table 2 for each enhancer according to employing equation1 and 2. The results obtained from administration of aminophylline containing gel without penetration enhancer into the membrane showed 70 ± 2.35% release after 3 hours. It is suggested that high permeability and drug solubility may effect on drug permeation through the membrane. Drug permeability through the skin was very low, and less than 1% of the drug was crossed after 6 hours. 

**Table 2.** Values of Flux (J), Permeability Coefficient (P), Enhancement Factor (EF), and Lag Time for Each Enhancer (n = 3)

Penetration Enhancer	J ± SD, mcg/cm^2^/h	P ± SD, 10^-6 ^cm/h	EF ± SD	Lag Time, h
**Control, group**	1.998 ± 0.28	99.90 ± 14.02	1	
**STGC, 100 μg**	12.83 ± 0.47	641.56 ± 23.37	6.42 ± 0.24	
**STGC, 200 μg**	9.23 ± 0.14	461.73 ± 67.76	4.62 ± 0.68	
**STGC, 500 μg**	4.79 ± 0.20	239.57 ± 9.92	2.41 ± 0.10	
**Lauric acid, 1.7%**	4.22 ± 0.05	210.93 ± 2.57	2.11 ± 0.03	
**Lauric acid, 15%**	8.72 ± 0.07	435.58 ± 3.29	4.36 ± 0.03	
**Ethanol, 60%**	13.22 ± 2.105	661.03 ± 105	6.61 ± 1.05	0.617

**Figure 1.**
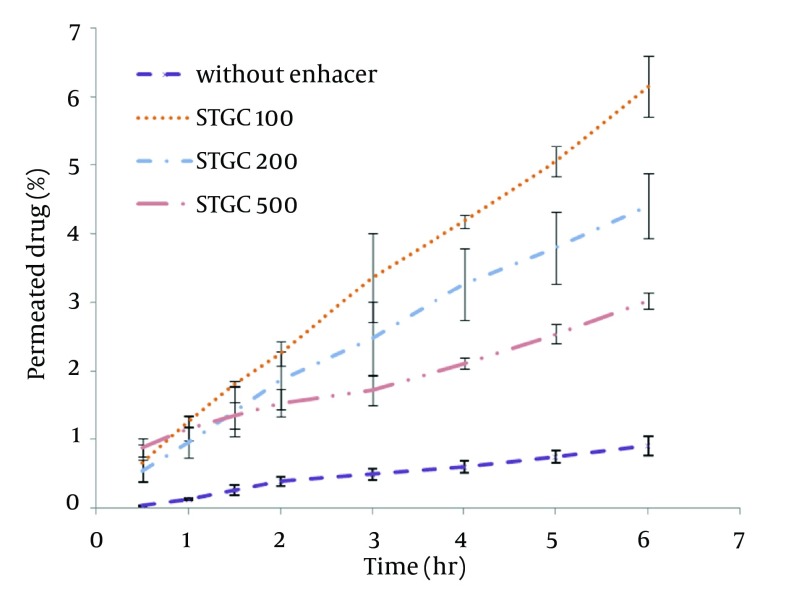
Aminophylline Skin Permeation from STGC Containing Gel as Penetration Enhancer

**Figure 2.**
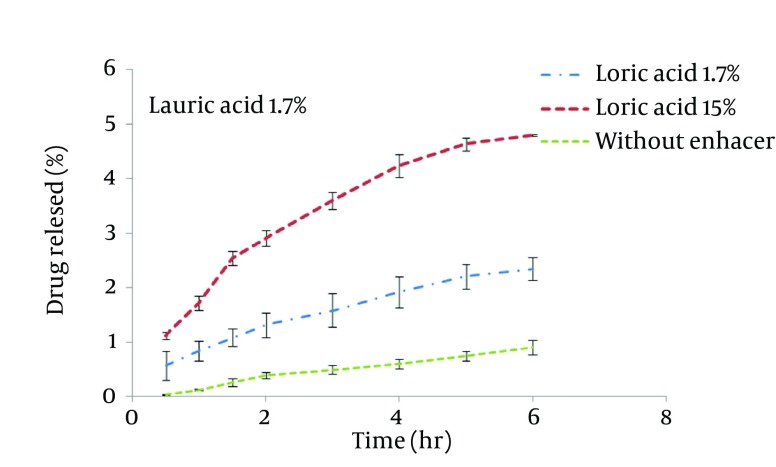
Aminophylline Skin Permeation from Lauric Acid Containing Gel as Penetration Enhancer

**Figure 3.**
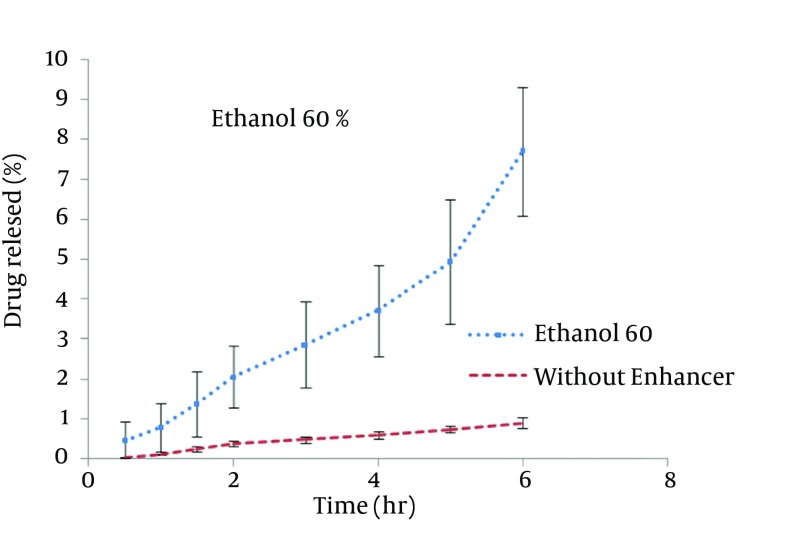
Aminophylline Skin Permeation from an Ethanol Containing Formulation as Penetration Enhancer

**Figure 4.**
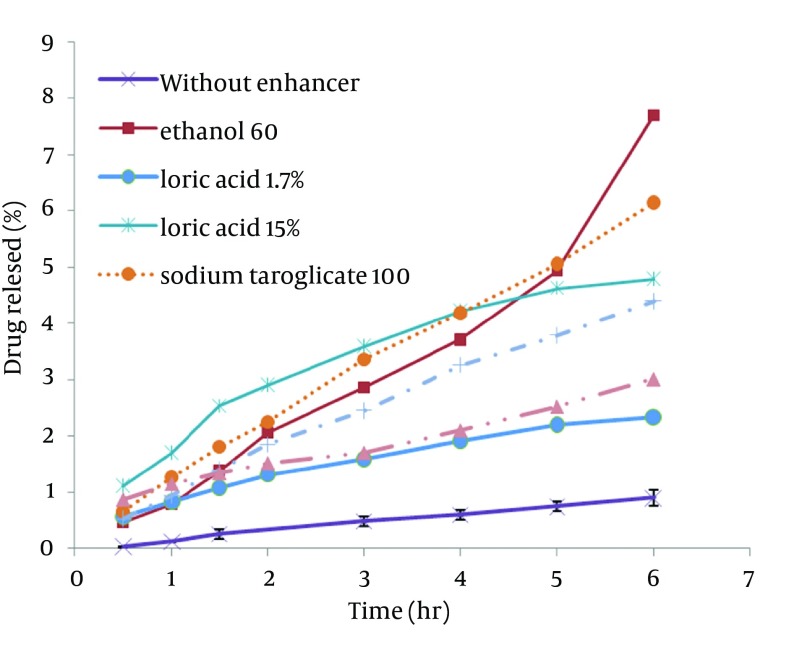
Permeation of Aminophylline Through Snake Shed Skin in the Presence of Different Absorption Enhancers

STGC improve the flux of aminophylline through the skin compared to the gel without enhancer (P < 0.05), and by increasing the concentration of enhancers, the permeation rate of the drug was significantly decreased (P > 0.05) (Figure 1). Based on Figure 2, lauric acid in both concentrations has increased EF value. This effect was significantly higher at concentration of 15% (P < 0.05). As shown in of Figure 3, ethanol (60%) significantly enhanced transdermal absorption of aminophylline through skin compared to control group. EF value was 13.22 ± 2.105. Figure 4 shows the comparison of transdermal permeation of drug from different formulations containing various enhancers. The results indicated that in the first hours, lauric acid (15%) and STGC (100 μg/mL) had the highest permeation effects, while after 4 hours it was higher for ethanol (60%), due to a lag time in drug absorption, showing that ethanol affected on the stratum corneum structure, gradually. The value of EF for ethanol (60%) and STGC (100μg/mL) were 5.94 ± 0.65 and 6.45 ± 0.24, respectively. 

## 5. Discussion

STGC is a surfactant used as a chemical permeation enhancer. In the study, it showed a significant improvement in the flux of aminophylline through the skin compared to the gel without enhancer (P < 0.05). It has been postulated that penetration of a surfactant into stratum corneum followed by interaction with and binding to keratin filaments leads to corneocyte disruption and surge of diffusion coefficient, which induce permeability. Also, many studies showed that surfactants may modify peptide or protein in the lipid bilayer stratum corneum that enhance permeability (14). In this study, by increasing the concentration of enhancers, the permeation rate of the drug was significantly decreased (P > 0.05) (Figure 1). It is suggested that when STGC concentration exceeds its critical micelle concentration (CMC), the drug entraps within the micelles and prohibits skin permeability. Our findings are in accordance with previous study that employed STGC as permeation enhancer for theophylline. According to the results, 2 hours after skin pretreatment with different concentration of STGC, the values of EF were shown as 50-105 in which the upper most value (EF = 105) was observed in concentration of 0.01%, while EF values were lower in higher concentrations (21). The studies of Errabelli et al. are in contradiction with our results. They investigated the STGC (5, 8, and 10%) as permeation enhancer for alfuzosin HCl using rat abdominal skin. The permeation was significantly improved which was concentration dependent. The EF values for STGC 5, 8 and 10% were 1.3, 1.4, and 1.8, respectively (22). However, in the present study, the EF values of this surfactant at concentrations of 0.01, 0.02, and 0.05% were 6.45, 4.65, and 2.41, respectively. Discrepancy observed in the results might be attributed to differences in physicochemical properties of drugs and type of skin models used in two studies. The enhancer effect of lauric acid, in both concentrations, has increased EF value. This effect was significantly higher at concentration of 15% (P < 0.05). On the other hand, diffusion across the skin was enhanced by increasing the concentration of permeation enhancers. EF values for lauric acid 1.7% and 15% were 2.12 ± 0.02 and 4.38 ± 0.03, respectively. A saturated alkyl chain with 10 to 20 atoms attached to polar groups causes appearance of strong effect of dermal absorption by fatty acid. These compounds change configuration structure of lipid chains, induce disorder, and decrease phase transition temperature of gel-liquid crystal (23-25). Propylene glycol was used as co-solvent for aminophylline in all gel formulations. Many studies have shown that the combination of propylene glycol with fatty acid has exhibited a synergistic effect on absorption enhancement. It is suggested that the enhancement might be due to facilitated incorporation of fatty acid into stratum corneum lipid alkyl domains through interaction of propylene glycol at polar head regions causing drug permeation through skin (26). It was previously shown that the combination of oleic acid and propylene glycol has increased absorption of tenoxicam in carbopol 940 gels (27). Similar results were obtained from investigations of Funke et al. in 2002. They reported that anti-estrogen drugs could permeate through skin by using the combination of propylene glycol and lauric acid (9:1) as absorption enhancer by which the increase of lauric acid concentration from 2.5 to 11% resulted in nearly 10 fold increase in transdermal delivery of this compound (28). Lauric acid shows high affinity to skin due to its optimal partition coefficient and solubility parameter (29). Wongpayapkul et al. in 2006 investigated the effect of various permeation enhancers on the skin permeation of ketoprofen. The results showed that lauric acid, capric acid, caprylic acid, and oleic acid were the most effective enhancers. Also, other researchers showed that in ketoprofen solution, lauric acid promoted penetration of ketoprofen through rat skin (30). In a research, isopropyl myristate as enhancer for permeability of naproxen through snake skin was studied. In the presence of absorption enhancer, the permeability of ionized form of naproxen across the membrane was significantly increased compared to non-ionized form. It was suggested that action mechanism of this fatty acid might be attributed to lipid disorder (31). As shown in Figure 3, ethanol (60%) significantly enhanced transdermal absorption of aminophylline through skin compared to control group. EF value was 13.22 ± 2.105. Krishnaiah et al. in 2002 reported that ethanol and water-ratio: 70/30 v/v-was effective in nicardipine hydrochloride absorption through rat skin (32). Ethanol influences on stratum corneum structure by reducing density of cross-linked bonds in intracellular and intercellular proteins. Also, it decreases area crossings and shortens the passage. Chemical materials such as propylene glycol and ethanol can replace water in protein chains and alter the arrangement of proteins from α-helix to sheet β (33, 34). Concentration of 60% was selected based on previous studies. It has been reported that the effect of ethanol as an absorption enhancer is concentration dependent. Pershing et al, in 1990 showed that using ethanol (60%), the estradiol flux across a human epidermal membrane increased but at higher concentrations, drug penetration was reduced (35). Several investigations have demonstrated that alcohol may increase the permeation of drugs by lipid extraction that occurs due to lipid solubilization, and increase the porosity of the stratum corneum leading to penetration of hydrophilic drug. The length of alkyl chain is important for skin penetration-enhancing action of alcohols so that by up to six carbon atoms, the permeation could be increased. Results of a study showed that the use of alcohols with higher carbon atom numbers decreased the penetration of indomethacin (27, 36). 

It can be concluded that the type and concentration of penetration enhancers are very important to achieve efficient transdermal delivery of drug. The results revealed that STGC (100 μg/mL), lauric acid (15%), and ethanol (60%) are potent absorption enhancers for aminophylline through shed snake skin. The enhancing effects of STGC (100 μg/mL) and ethanol (60%) were the same but the penetrating process for formulations containing STGC (100 μg/mL) was started without delay.
